# Upregulation of spondin-2 predicts poor survival of colorectal carcinoma patients

**DOI:** 10.18632/oncotarget.3822

**Published:** 2015-04-14

**Authors:** Qian Zhang, Xiao-Qing Wang, Jie Wang, Shu-Jian Cui, Xiao-Min Lou, Bing Yan, Jie Qiao, Ying-Hua Jiang, Li-Jun Zhang, Peng-Yuan Yang, Feng Liu

**Affiliations:** ^1^ Department of Systems Biology for Medicine, School of Basic Medical Sciences, and Institutes of Biomedical Sciences, Fudan University, Shanghai, China; ^2^ Department of Chemistry, Fudan University, Shanghai, China; ^3^ Minhang Hospital, Fudan University, Shanghai, China; ^4^ Shanghai Public Health Clinical Center, Fudan University, Jinshan District, Shanghai, China; ^5^ College of Bioscience and Biotechnology, Key Laboratory of Crop Genetics and Physiology of Jiangsu Province, Yangzhou University, Yangzhou, Jiangsu, China; ^6^ CAS Key Laboratory of Genome Sciences and Information, Beijing Institute of Genomics, Chinese Academy of Sciences, Chaoyang District, Beijing, China; ^7^ Key Laboratory of Digestive Organ Transplantation of Henan Province and the Department of Hepatobiliary and Pancreatic Surgery, The First Affiliated Hospital of Zhengzhou University, Zhengzhou, Henan, China

**Keywords:** colorectal cancer, SPON2, spondin-2, prognosis, protein marker

## Abstract

Colorectal cancer (CRC) is the third and second most common cancer in males and females worldwide, respectively. Spondin-2 is a conserved secreted extracellular matrix protein and a candidate cancer biomarker. Here we found that *Spondin-2* mRNA was upregulated in CRC tissues using quantitative RT-PCR and data-mining of public Oncomine microarray datasets. Spondin-2 protein was increased in CRC tissues, as revealed by immunohistochemistry analyses of two tissue microarrays containing 180 cases. *Spondin-2* gene expression was significantly associated with CRC stage, T stage, M stage and Dukes stage, while its protein was associated with age and M stage. Kaplan-Meier analysis revealed that the upregulated *Spondin-2* mRNA and protein predicted poor prognosis of CRC patients. Univariate and multivariate Cox regression analyses indicated that grade, recurrence, N stage and high Spondin-2 were independent predictors of overall survival of CRC patients. ELISA revealed that plasma Spondin-2 was upregulated in CRC and dropped after surgery. Receiver operating characteristic curve analysis demonstrated that plasma Spondin-2 has superior predictive performance for CRC with an area under the curve of 0.959 and the best sensitivity/specificity of 100%/90%. Furthermore, ectopic expression of Spondin-2 enhanced colon cancer cell proliferation. Spondin-2 could be an independent diagnostic and prognostic biomarker of colon cancer.

## INTRODUCTION

Colorectal cancer (CRC) is the third most common cancer in males and the second in females worldwide. According to the latest GLOBOCAN 2012 statistics, there were 746 thousand cases in men and 614 thousand cases in women [1]. The overall mortalities are 50.1% (374 thousand) and 52.1% (320 thousand) in men and women, respectively, indicating a very poor prognosis of this deadly cancer. More than half of the total cases are occur in economically developed countries. In China, the estimated cases of CRC in 2012 is 253 thousand, and the predicted mortality is 55% (139 thousand) [1]. Smoking [2], alcohol consumption, age [3], heredity, lower calcium intake and obesity [4] have been recognized as potential risk factors of CRC. Whereas the high dietary fiber intake is considered to be helpful to reduce the risk of CRC [5]. The genetic predispositions, such as chromosomal instability, microsatellite instability and epigenetic alterations like CpG island methylation, have been revealed to be the major contributors to CRC tumorigenesis [6]. Despite the achievements that having been made to date, the radical cure of CRC still remains a challenge task due to the heterogeneity and complexity of CRC, the drug resistance during treatment, and the recurrence and metastasis after surgery [7]. A precise diagnosis at an early stage and an accurate evaluation of post-operative survival would greatly increase the chances for a successful treatment. Therefore, there is an argent need to identify suitable diagnostic or prognostic molecular biomarkers, which may be helpful for cancer identification and therapy.

*Spondin-2* (*SPON2*), encoding a secretory protein and an conserved extracellular matrix (ECM) protein with homology to the mindin/F-spondin family, was first cloned from noncancerous lung cells and found to be downregulated in cancerous lung cells [8]. SPON2 is a host innate immune regulator and represents a pattern-recognition molecule for microbial pathogens [9]. Increased *SPON2* gene and protein expression has been observed in liver cancer [10, 11], gastric cancer [12], ovarian cancer [13, 14], pancreatic cancer [15], breast cancer [16], Barrett's adenocarcinoma [17] and prostate cancer [18-23]. In addition, *SPON2* gene was upregulated in colorectal carcinoma comparing with colorectal adenoma [24, 25]. SPON2 has been proposed as a diagnostic biomarker of prostate cancer [19-23] and ovarian cancer [13, 18, 26, 27]. The expression of SPON2 in human cancers might be modulated by hormones, like thyroid hormone [10, 28] or androgen [21], as well as by epigenetic mechanism [20, 29].

Despite above findings, the relationships of *SPON2* mRNA and protein overexpression with clinicopathological parameters and prognosis of colon cancer remain further explorations. In current study, we analyzed the mRNA expression level of *SPON2* in CRC tissues samples using quantitative reverse-transcriptional PCR (qRT-PCR) and public Oncomine microarray datasets. Furthermore, we measured the expression of SPON2 protein in CRC using two commercial tissue microarrays (TMAs) and immunohistochemistry (IHC). The protein expression of SPON2 in the colon cancer cell lines and plasma of CRC patients were analyzed using Western blot and enzyme-linked immunosorbent assay (ELISA), respectively. The associations of SPON2 expression with the clinicopathological factors and the survival of CRC patients were assessed using Kaplan-Meier analysis and Cox proportional hazards modeling. We also performed receiver operating characteristic (ROC) curve analysis to address the predictive performance of SPON2 in diagnosis of CRC. Finally, we performed an overexpression analysis of SPON2 to address the functional role of SPON2 in CRC cells. Our analysis suggested that SPON2 could be an independent diagnostic and prognostic biomarker of colon cancer.

## RESULTS

### SPON2 mRNA and protein were significantly upregulated in CRC

**Figure 1:**
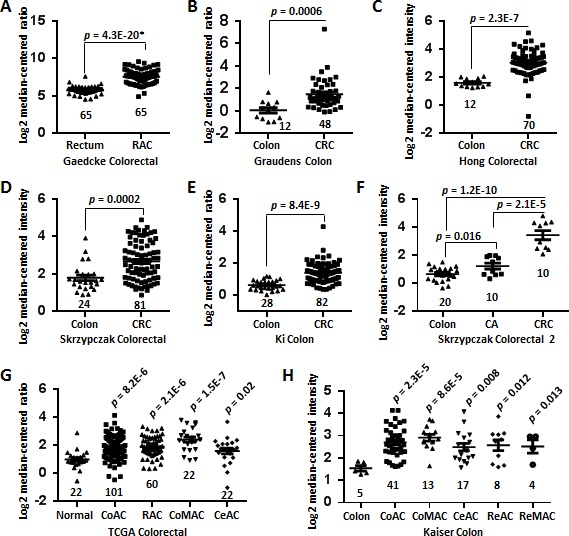
Upregulation of SPON2 mRNA in CRC revealed by data-mining of the Oncomine gene expression database **A.** Gene expression of *SPON2* is upregulated in rectal adenocarcinoma (RAC) tissues comparing with the normal rectum tissues revealed using the Gaedcke Colorectal dataset from Oncomine database (https://www.oncomine.org/resource/login.html). **B.** Differential *SPON2* gene expression in the normal colon and colorectal carcinoma (CRC) tissues revealed by the Graudens Colon dataset. **C.** *SPON2* expression in the colon and CRC specimens in the Hong Colorectal dataset. **D.** *SPON2* expression analysis in the colon and CRC tissues in the Skrzypczak Colorectal dataset. **E.** *SPON2* expression in the normal colons and CRCs in the Ki Colon dataset. **F.** *SPON2* expression in the colon, colon adenoma (CA) and CRC tissues in the Skrzypczak Colorectal 2 dataset. **G.** *SPON2* expression in the normal tissues (colon or rectum), colon adenocarcinoma (CoAC), RAC, colon mucinous adenocarcinoma (CoMAC), cecum adenocarcinoma (CeAC) and CRC tissues in the TCGA colorectal dataset. **H.** *SPON2* expression in the colon, CoAC, CoMAC, CeAC, rectosigmoid adenocarcinoma (ReAC), rectal mucinous adenocarcinoma (ReMAC) tissues in the Kaiser Colon dataset. The total number of cases were shown under each categories. The *p* values were calculated using two-tailed and unpaired Student's *t* test. The *p* value indicated with an asterisk was calculated using paired Student's *t* test.

We analyzed the differential expression of *SPON2* mRNA between colon cancer tissues and normal colonic tissues by data-mining of the Oncomine microarray gene expression datasets. We found that *SPON2* expression was significantly upregulated in rectal adenocarcinoma tissues comparing with paired normal colonic rectum tissues using Gaedcke Colorectal dataset tissues (*n* = 65, *p* = 4.3E-20, paired Student's *t* test) (Figure 1A). Expression of *SPON2* was also significantly higher in the CRC tissues than the normal colon tissues by using Graudens Colon dataset (Figure 1B, *n* = 48, *p* = 0.0006), Hong Colorectal dataset (Figure 1C, *n* = 70, *p* = 2.3E-7), Skrzypczak Colorectal dataset (Figure 1D, *n* = 81, *p* = 0.0002) and Ki Colon dataset (Figure 1E, *n* = 82, *p* = 8.4E-9). Interestingly, *SPON2* mRNA was gradually elevated from colon adenoma to CRC (*n* = 10, *p* = 2.1E-5) by analyzing Skrzypczak Colorectal 2 dataset (Figure 1F). This observation highlighted a potential role of *SPON2* in the tumorigenesis or malignancy of colon cancer. Furthermore, *SPON2* was found to be significantly elevated in every types of colon cancers, including colon adenocarcinoma (*n* = 101, *p* = 8.2E-6), rectal adenocarcinoma (*n* = 60, *p* = 2.1E-6), colon mucinous adenocarcinoma (*n* = 22, *p* = 1.5E-7) and cecum adenocarcinoma (*n* = 22, *p* = 0.02), as revealed in TCGA Colorectal dataset (Figure 1G). This observation was confirmed by another independent dataset, Kaiser Colon, where *SPON2* was upregulated in all five types of colorectal cancers comparing with normal colon mucosa tissues (*n* = 4 ~ 41, *p* = 0.012 ~ 2.3E-5) (Figure 1H). It should be noted that these analyses, which showed a consistent upregulation of *SPON2* in colon cancers, were performed independently and contained a total of 654 cancer cases and 178 normal controls.

**Figure 2:**
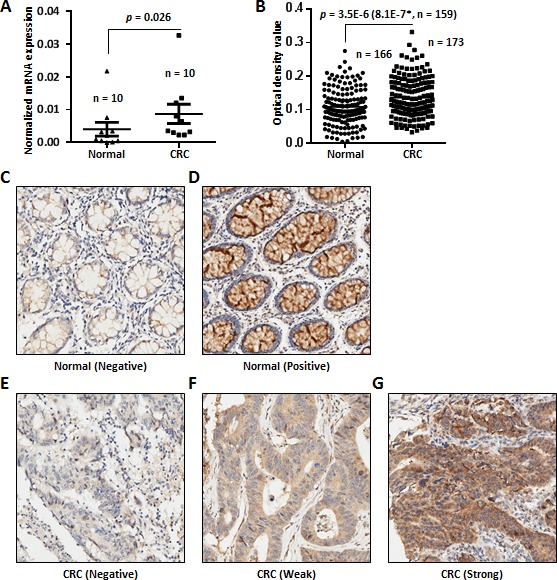
SPON2 mRNA and protein expression in CRC tissues **A.** *SPON2* expression was evaluated in 10 pairs of CRC tissue samples and the matched normal counter parts. The *p* values were calculated by paired Student's *t* test and a *p* value < 0.05 was considered as statistically significant. **B.** Expression of SPON2 in colon cancer tissues and paired normal mucosa counterparts revealed by IHC analysis using two commercial TMAs. One TMA (HCol-Ade180Sur-04, Shanghai Outdo Biotech, China) contains 90 paired normal and CRC tissues with follow-up information. Another commercial TMA (HCol-Ade180CS-01, Shanghai Outdo Biotech, China) contains 90 cases without follow-up information. The scoring of SPON2 protein expression between the normal and cancer tissues was based on the scanned staining intensity values analyzed using Image Pro Plus 6.0. The *p* value was calculated by unpaired and two-sided Student's *t* test and the *p* value in the bracket was calculated using paired and two-sided Student's *t* test for 159 paired cases. A *p* value < 0.05 was considered as statistically significant. The cases without detectable epithelial cells in the chip were excluded from analysis. **C.** Negative SPON2 expression in a normal colonic mucosa specimen. **D.** Positive staining of SPON2 in a normal colonic mucosa specimen. **E.** Negative SPON2 staining in a CRC specimen. **F.** Low expression of SPON2 in a CRC specimen. **G.** High expression of SPON2 in a CRC specimen.

To confirm the above findings, we performed a qRT-PCR analysis of *SPON2* expression in CRC tissues. As shown in Figure 2A, *SPON2* mRNA was significantly increased in CRC tissues comparing with the matched normal mucosa counterparts (*n* = 10, *p* = 0.026).

**Figure 3:**
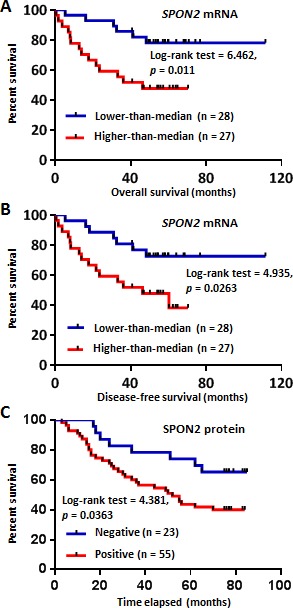
Kaplan-Meier analysis of SPON2 expression in CRC patients based on the Oncomine dataset mining and TMA-IHC analysis **A.** Overall survival analysis of CRC patients with different *SPON2* gene expression using Smith Colorectal 2 dataset from Oncomine database. The low or high expression of *SPON2* was defined as lower-than the median or higher-than the median. **B.** Disease-free survival analysis of CRC patients with different *SPON2* expression using Smith Colorectal 2 dataset. **C.** Positive SPON2 protein expression in CRC patients predicted poor prognosis as revealed by TMA-IHC analysis. The commercial TMA contains 90 CRC cases, while those cases without follow-up information and/or without detectable epithelial cells were excluded from the analysis. The *p* value was calculated using the Log-rank (Mantel-Cox) test.

To address the protein change of SPON2 in colon cancer tissues, we performed IHC analyses of SPON2 expression using two commercial independent TMAs, each of which contains 90 cases of colorectal adenocarcinoma. The stained TMAs were scanned and images of each cases were extracted and subjected to the analysis of mean integrated optical density (IOD) using Image Pro Plus. Only the IOD of cancer epithelial cells in each images were analyzed and the values from different area and different images of the same sample were averaged. Some samples were excluded from analysis due to the absence of visible epithelial cancer cells. The expression of SPON2 protein was substantially upregulated in CRC tissues (*n* = 173) comparing with the normal counterparts (*n* = 166) (unpaired Student's *t* test, *p* = 3.5E-6) (Figure 2B). The difference was more significant by comparing 159 paired normal and cancer samples (paired, two-sided Student's *t* test, *p* value = 8.1E-7). The staining of negative and positive SPON2 expression in the normal colonic mucosa tissues were shown in Figure 2C and 2D, respectively. The negative staining of SPON2 of one CRC tissue sample was depicted in Figure 3E. In addition, the typical weak and strong SPON2 staining of CRC tissues were displayed in Figure 3F and 3G, respectively.

### Association of SPON2 mRNA and protein expression with clinicopathological characteristics of CRC patients

We analyzed the relationship of *SPON2* mRNA level with the clinicopathological characteristics of CRC patients using available Oncomine datasets. For each dataset, the cases was divided into two groups based on the median expression value of *SPON2*. Expression of *SPON2* was significantly associated with overall stage (Bittner Colon, χ^2^= 16.96, *p* = 0.001), T stage (Bittner Colon, χ^2^= 13.852, *p* = 0.003), M stage (Bittner Colon, χ^2^= 3.967, *p* = 0.046), Dukes stage (Bittner Colon, χ^2^= 13.514, *p* = 0.001; Jorissen Colorectal 3, χ^2^= 11.337, *p* = 0.01) and years of tobacco consumption (Bittner Colon, Fisher's Exact Test, *p* = 0.047) (Table 1). Analyses of two datasets indicated that *SPON2* was significantly higher in male patients than in females (Gaedcke Colorectal, χ^2^= 8.021, *p* = 0.005; Tsuji Colorectal, χ^2^= 3.967, *p* = 0.046) (Table 1).

**Table 1:** Correlation between the mRNA expressions of SPON2 and clinicopathological variables in patients with colorectal cancer revealed by data-mining of Oncomine gene array datasets

Dataset^a^	Clinicopathological parameters	Total cases	SPON2 expression		χ^2^	*p* value
			Low-than-median	High-than-median		
Bittner colon						
	Dukes Stage					
	A	49	34	15	13.514	0.001
	B	101	43	58		
	C-D	87	33	54		
	M stage					
	M0	240	112	128	3.976	0.046
	M1	88	52	36		
	Stage					
	0-I	52	37	15	16.96	0.001
	II	103	42	61		
	III	85	37	48		
	IV	88	52	36		
	T stage					
	Tis-T1	15	12	3	13.852	0.003
	T2	55	35	20		
	T3	220	95	125		
	T4	28	12	16		
	Years of Tobacco Use					
	None	17	4	13	Fisher's Exact Test	0.047
	0~65	187	89	98		
Jorissen Colorectal 3						
	Dukes Stage				11.337	0.01
	A	24	18	6		
	B	54	28	26		
	C	43	14	29		
	D	33	17	16		
Gaedcke Colorectal						
	Sex				8.021	0.005
	Male	44	17	27		
	Female	21	16	5		
Tsuji Colorectal						
	Sex				3.967	0.046
	Male	54	23	31		
	Female	29	19	10		

a The analysis was performed using datasets from the Oncomine cancer gene expression microarray DB (https://www.oncomine.org/resource/login.html).

The IHC analyses of two commercial TMAs provided additional information of the relationship of SPON2 expression with clinicopathological parameters of CRC patients. To increase the statistic power of the analysis of SPON2 protein expression in CRC patients, we combined the IHC results from two TMAs with a total of 180 different cases enrolled. We found that SPON2 protein expression was remarkably related to age (χ^2^= 9.65, *p* = 0.0468) and M stage (χ^2^= 4.245, *p* = 0.039) of CRC patients (Table 2). In addition, the relationship of SPON2 expression with stage was at the margin of statistical significance (χ^2^= 7.446, *p* = 0.059).

**Table 2:** Correlation of SPON2 protein expression with clinicopathological variables in CRC patients revealed by TMA-IHC analysis

Clinicopathological factor		Total cases	SPON2 (−)	SPON2 (+)	χ2	*p* value
Gender						
	Male	98	37	61	0.522	0.47
	Female	74	24	50		
Age						
	< 50	12	9	3	9.65	0.0468
	50-59	27	9	18		
	60-69	57	16	41		
	70-79	50	18	32		
	≥ 80	23	9	14		
Grade						
	I	7	4	3	2.212	0.331
	II	110	41	69		
	III	56	17	39		
Vascular invasion present						
	Positive	24	9	15	0.745	0.388
	Negative	37	10	27		
Lymphatic invasion present						
	Positive	54	26	28	Fisher's Exact Test	0.487
	Negative	3	2	1		
Number of positive lymph nodes						
	< 5	142	45	97	1.129	0.288
	≥ 5	13	6	7		
T stage						
	T1-T2	25	6	19	1.887	0.3892
	T3	107	41	66		
	T4	36	12	24		
N stage						
	N0	96	31	65	1.405	0.495
	N1	52	20	32		
	N2	25	11	14		
M stage						
	M0	155	52	103	4.245	0.039
	M1	17	10	7		
Stage						
	1	23	4	19	7.446	0.059
	2	67	25	42		
	3	64	22	42		
	4	17	10	7		
Metastasis						
	Yes	156	52	104	4.332	0.037
	No	17	10	7		
Tumor volume						
	< 100 cm^3^	141	52	89	0.227	0.634
	≥ 100 cm^3^	28	9	19		

### Upregulated SPON2 mRNA and protein predicts poor prognosis of CRC patients

First, we tried to determine whether the overexpression of *SPON2* mRNA associates with the prognosis of CRC patients or not. Smith Colorectal 2 dataset in the Oncomine database contains 55 cases, including 20 completed cases and 35 censored cases. The patients were divided into lower-than-median and higher-than-median expression group based on the Log2 median-centered intensity value of *SPON2* mRNA. We performed Kaplan-Meier analysis of the relationship of *SPON2* expression with the survival of the CRC patients. In the analysis of overall survival of CRC patients, we found that high expression of *SPON2* mRNA was significantly associated with poor prognosis of the CRC patients (Log-rank test = 6.462, *p* = 0.011) (Figure 3A). We also analyzed the relationship of *SPON2* expression with the disease-free survival of CRC patients and found that upregulation of *SPON2* also predicted worse outcome of CRC patients (Log-rank test = 4.935, *p* = 0.026) (Figure 3B).

We further analyzed the relationship of the protein expression of SPON2 with the prognosis of CRC patients using the commercial TMA HCol-Ade180Sur-04 which contains follow-up information. The Kaplan-Meier survival analysis revealed that the positive expression of SPON2 protein in cancerous epithelial cells indicated a worse prognosis of CRC patients (Log-rank test = 4.381, *p* = 0.036) (Figure 3C).

These results suggested that both the *SPON2* mRNA and protein expression could be potential indicators of CRC prognosis.

### Univariate and multivariate Cox regression analyses of clinical variables of CRC patients

We performed univariate and multivariate Cox regression analyses of *SPON2* mRNA or protein expression and clinical variables for overall survival of CRC patients. By univariate analysis using the Smith Colorectal 2 dataset in Oncomine database, we revealed that recurrence (Hazard ratio (HR) = 9.462, 95% confidence interval (CI) = 3.395-26.368, *p* < 0.001), high *SPON2* expression (HR = 3.245, 95% CI = 1.243-8.473, *p* =0.016) and high level stage (HR = 2.972, 95% CI = 1.562-5.654, *p* = 0.001) were hazard factors for overall survival of CRC patients (Supplementary Table 1). While grade was at the margin statistical significance (HR = 4.588, 95% CI = 0.993-21.2, *p* = 0.051). Using multivariate analysis, grade (HR = 8.595, 95% CI = 1.404-52.599, *p* = 0.020) and recurrence (HR = 7.857, 95% CI = 1.722-35.859, *p* = 0.008) were found to be independent factors affecting overall survival.

By the TMA-IHC assays, we identified in univariate analyses that high T stage (HR = 2.332, 95% CI = 1.267-4.291, *p* = 0.007), N stage (HR = 2.530, 95% CI = 1.673-3.825, *p* = 0.000), high grade (HR = 2.470, 95% CI = 1.071-5.695, *p* = 0.034) and high SPON2 protein expression (HR = 1.860, 95% CI = 1.298-2.665, *p* = 0.001) were risk factors of overall survival of CRC patients (Table 3). Using multivariate Cox regression method, we revealed that SPON2 protein expression (HR = 1.786, 95% CI = 1.249-2.555, *p* = 0.001) and high N stage (HR = 2.554, 95% CI = 1.655-3.940, *p* = 0.000) were hazard factor of overall survival of CRC patients (Table 3).

These results indicated that high SPON2 protein expression could be an independent diagnostic and prognostic marker of CRC patients.

**Table 3:** Univariate and multivariate Cox regression analyses of SPON2 protein expression and clinical variables for overall survival of CRC patients

Methods	Variables	β	SE (β)	Wald χ^2^	*p*	Hazard ratio	95% CI	
							Low	Upper
Univariate Cox regression								
	Gender	−.277	.298	.867	.352	.758	.423	1.358
	Age	.010	.012	.725	.395	1.010	.987	1.034
	T stage	.847	.311	7.405	.007	2.332	1.267	4.291
	N stage	.928	.211	19.373	.000	2.530	1.673	3.825
	M stage	.697	.724	.926	.336	2.007	.486	8.290
	Grade	.904	.426	4.502	.034	2.470	1.071	5.695
	SPON2 expression	.620	.184	11.425	.001	1.860	1.298	2.665
Multivariate Cox regression								
	N stage	.938	.221	17.958	.000	2.554	1.655	3.940
	SPON2 expression	.580	.183	10.096	.001	1.786	1.249	2.555

β, regression coefficient; SE, standard error; CI, confidence interval.

### Secreted SPON2 protein as a plasma marker of CRC

**Figure 4:**
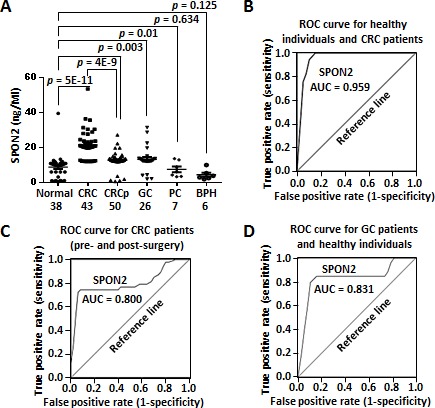
Secreted SPON2 protein as a plasma marker of CRC **A.** The protein level of secreted SPON2 were evaluated using the ELISA method. CRC, colorectal cancer; CRCp, colorectal cancer (postoperative); GC, gastric cancer; PC, prostate cancer; BPH, benign prostatic hyperplasia. Case numbers were listed under each category. *p* value was calculated using unpaired, two-sided Student's *t* test. **B.** ROC curve analysis of plasma SPON2 in the CRC patients (*n* = 43) and the healthy individuals (*n* = 38). AUC, area under curve. **C.** ROC curve analysis of plasma SPON2 in the pre- (*n* = 43) and post-surgery CRC patients (*n* = 50). **D.** ROC curve analysis of plasma SPON2 in the GC patients (*n* = 26) and the healthy controls.

SPON2 is a secreted protein and we were interested in its diagnostic potential as a plasma biomarker for CRC. We performed a sandwich ELISA assay of SPON2 expression in the plasma samples using pre-coated 96-well strip plates. The plasma SPON2 was significantly higher in the CRC patients (*n* = 43) than in the healthy peoples (*n* = 38) (*p* = 5E-11) (Figure 4A). Interestingly, SPON2 concentration was dropped significantly after surgery (*n* = 50, *p* = 4E-9), although it was still higher than that in the healthy controls. The concentration of SPON2 in the plasma of gastric cancer patients were low comparing with that of CRC patients, but was also higher than that of the healthy individuals. The plasma concentrations of SPON2 in the prostate cancer patients (*n* = 7, *p* = 0.634) and benign prostatic hyperplasia patients (*n* = 6, *p* = 0.125) tested here had no significant differences comparing with that of the healthy donors. The average concentrations of SPON2 in the plasma of healthy donors, CRC patients, CRC patients after surgery, gastric cancer (GC) patients, prostate cancer patients and benign prostatic hyperplasia patients were 8.8, 21.6, 12.7, 13.4, 7.6 and 4.5 ng/mL, respectively.

We then performed a ROC curve analysis of the plasma concentration of SPON2 in the healthy donors and CRC patients. When all 43 CRC cases were compared against all 38 healthy controls, SPON2 showed an area under the curve (AUC) of 0.959, which indicated a strong capability to distinguish CRC cases from healthy controls (Figure 4B). At a concentration of 12.1 ng/mL, SPON2 showed the best sensitivity of 100% and the best specificity of 90%.

Furthermore, we analyzed the ROC curve of SPON2 in pre- and post-surgery CRC patient samples. Interestingly, the plasma SPON2 displayed a strong power to discriminate tumor-free patients from tumor-bearing patients (AUC = 0.800) (Figure 4C). The best sensitivity of 74.4% and the best specificity of 92% were found at a concentration of 18.2 ng/mL.

We also analyzed the performance of plasma SPON2 in discrimination of the healthy individuals and GC patients. The AUC value was 0.831 and the best sensitivity and specificity (at 12.1 ng/mL) were 84.6% and 89.5%, respectively, indicating a good diagnostic performance of SPON2 for GC.

### Elevated SPON2 protein contributes to accelerated proliferation of colon cancer cells

**Figure 5:**
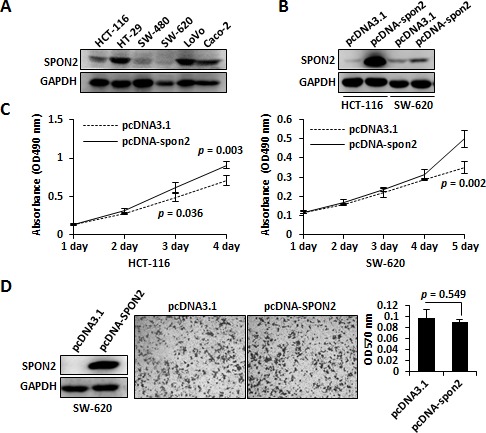
The functional role of SPON2 in colon cancer cell lines **A.** SPON2 protein levels in colon cancer cell lines were analyzed using Western blot. **B.** *SPON2* was overexpressed in colon cell lines HCT-116 and SW-620 and the expression level of SPON2 was analyzed using Western blot. **C.** Growth curve analysis of SPON2 ectopic expression in colon cancer cells was performed using the MTT method. The *p* values were calculated using replicated data by Student's *t* test and *p* < 0.05 was considered as statistically significant. **D.** The SPON2 was overexpressed in SW-620 cell and the migration was measured using Transwell method. The cells migrated to the bottom of the membrane were stained with crystal violet and the dye was dissolved using acetic acid and absorbance was measured at 570 nm.

To get further insight into the functional role of SPON2 in tumorigenesis of colon cancer, we performed *in vitro* proliferation assays using colon cancer cell lines. Western blot analysis of SPON2 expression indicated that SPON2 was abundant in colon cancer cell lines HT-29, LoVo and Caco-2, but low in HCT-116, SW-480 and SW-620 (Figure 5A). We then overexpressed the full-length *SPON2* gene in HCT-116 and SW-620 (Figure 5B). The proliferation of both cells were measured using methylthiazolyldiphenyl-tetrazolium bromide (MTT)assay, which revealed that the upregulated SPON2 induced an significantly accelerated proliferation of HCT-116 and SW-620 (Figure 5C). We also analysed the migration potential of SW-620 cells with ectopic expressed SPON2. However, no significant change was observed after SPON2 expression (Figure 5D).

## DISCUSSION

SPON2 is a secreted ECM protein belonging to the mindin/F-spondin family, which contains a large spondin domain and a C-terminal thrombospondin 1 domain. Spondin proteins were reported to play important roles in cancerous signaling. For example, R-spondin was involved in the activation of WNT signaling and its recurrent gene fusion events had been addressed in colon cancer [30, 31]. The biological function of SPON2 remains largely unknown. The upregulation of *SPON2* gene has been detected in liver cancer [10, 11], gastric cancer [12], ovarian cancer [13, 14], pancreatic cancer [15], breast cancer [16], Barrett's adenocarcinoma [17] and prostate cancer [18-23]. SPON2 has been raised as a potential clinical biomarker for prostate and ovarian cancer.

In current study, we analyzed SPON2 expression in colon cancer cell lines, CRC clinical tissues and CRC plasma samples and revealed that SPON2 mRNA and protein were upregulated significantly in CRC specimens comparing with normal colonic samples. The increased *SPON2* mRNA expression in CRC was revealed by data mining of eight independent microarray datasets in the Oncomine database, with a total of 654 cancer samples and 178 normal controls analyzed. An interesting finding was that *SPON2* was upregulated in colorectal carcinoma comparing with adenoma, which was in line with previous findings [24, 25]. These observations suggested that SPON2 might play a role during the carcinogenesis or malignancy of colon cancer. We verified the upregulation of *SPON2* mRNA in CRC tissues by qRT-PCR. We also examined the protein expression of SPON2 in CRC tissues by IHC analyses of a total of 180 CRC cases using TMAs and revealed that SPON2 protein was significantly upregulated in CRC tissues comparing with the normal mucosa counterparts.

The relationship of SPON2 expression with clinicopathological parameters of colon cancer was less addressed. The upregulation of SPON2 protein has been verified in hepatocellular carcinoma tissues by IHC method, while the expression was not found to be correlated with pathological stages [10]. Here, we identified that the increased *SPON2* mRNA expression was significantly associated with stage, T stage, M stage, Dukes stage and tobacco consumption time of CRC patients using Oncomine datasets. Furthermore, our IHC analyses indicated that SPON2 expression was significantly related to age and M stage. The association of SPON2 expression with grade was at the margin of statistical significance (*p* = 0.059). These observations suggest that SPON2 could serve as a histological diagnostic biomarker.

Several reports indicated that SPON2 expression might associate with cancer metastasis. *SPON2* mRNA was found to be upregulated in metastatic lymph node tumors comparing with primary breast cancer [16], as well as in invasive carcinoma comparing with *in situ* carcinoma of breast cancer [32]. *SPON2* mRNA was also significantly increased in high metastatic oral cancer cell line comparing with the parental cell line [33]. Although SPON2 protein was upregulated in hepatocellular cancer cell, *in vitro* assay indicated that SPON2 expression was negatively affected cell invasiveness and migration [10]. In prostate cancer, the mRNA of *SPON2* was reduced in lymph node metastasis cancer comparing with the recurrent or nonrecurrent primary cancer [21]. In current study, no significant change of colon cancer cells in migration was observed when SPON2 was overexpressed. These contradictory results suggested that the functional role of SPON2 in cancer metastasis should be further investigated.

The expression of SPON2 could be regulated epigenetically or by hormone. *SPON2* expression was induced by the thyroid hormone [10, 28] or androgen [21] in human cancer. Hypomethylation of *SPON2* promoter lead to an upregulation of *SPON2* in prostate cancer and meningioma [20, 29]. On the contrary, middle or high methylation of *SPON2* promoter resulted in low expression of *SPON2*, which was strongly correlated with favorable outcome of patients with acute lymphoblastic leukemia [34]. These observations suggested that cancerous *SPON2* gene expression was modulated at the transcription level. It also indicated that the expression alteration of *SPON2* might associate with prognosis of cancer patients.

To address the significance of *SPON2* in prognosis, we performed Kaplan-Meier survival analyses and found that high expressions of *SPON2* mRNA and protein were associated with adverse prognosis of CRC patients. The univariate Cox regression analysis indicated that elevated *SPON2* expression closely related to decreased overall survival of CRC patients. By IHC analysis, we found high SPON2 protein expression predicted poor outcome of CRC patients. The univariate and multivariate Cox regression analyses suggested that the elevated SPON2 expression might be a hazard factor for the survival of CRC patients. This observation was consistent with the previous finding that the upregulated serum SPON2 was associated with worse overall survival of ovarian cancer patients [27]. These results suggested both the mRNA and protein expression of SPON2 could serve as prognosis indicators of CRC.

As a secreted protein, serum SPON2 has been raised as a biomarker in human cancers, especially in prostate cancer [21-23] and ovarian cancer [13, 18, 26, 27]. In prostate cancer, the serum SPON2 achieved a predictive AUC value of 0.942 and a serum concentration of 8 ng/mL provided the best sensitivity of 100% and the best specificity of 84.6% [22] (Supplementary Table 2). In another report, the AUC of SPON2 was 0.952, and the sensitivity and specificity were 86% and 100% at 36 ng/mL, respectively [23]. In our analysis, the plasma SPON2 showed a high prediction value of AUC 0.959 and a highest sensitivity of 100% together with best specificity of 90% at a concentration of 12.1 ng/mL. Such results suggested that SPON2 had an equal or better performance in colon cancer than in prostate cancer. We also measured the plasma concentration of SPON2 in the plasma of prostate cancer and benign prostatic hyperplasia, while no significant differences were observed comparing with the healthy donors, due possibly to the limited number of samples analyzed. In addition, for the first time, we analyzed the diagnostic performance of SPON2 in GC patients. The performance in GC (AUC = 0.831, sensitivity 84.6%, specificity 89.5%) was also good comparing with that in ovarian cancers, where the AUCs ranging from 0.58 ~ 0.84 [13, 14, 26]. These findings indicated that SPON2 might not be the prostate cancer-specific diagnostic biomarker [22]. Even though, an upregulation of plasma SPON2 would be an indication of malignancies. The higher the plasma concentration of SPON2, the more likely that it might be a colon cancer.

An interesting finding would be that the plasma SPON2 of the CRC patients (average = 21.6 ng/mL) was downregulated after surgery (average = 12.7 ng/mL), suggesting that SPON2 was closely associated with tumor burden. This observation indicated that plasma SPON2 could be useful for operation evaluation.

In colon cancer, as an single independent diagnostic or prognostic biomarker, the performance of SPON2 might be equivalent or better than some raised biomarkers, such as carcino-embryonic antigen (CEA) (AUC = 0.856, sensitivity 74%) [35], cancer antigen (CA) 19-9 (AUC = 0.58, sensitivity 26%) [35], dipeptidase 1 (AUC 0.923) [36], interleukin-8 (AUC = 0.742, sensitivity 85.4%/specificity 54%) [37], miR-21 (AUC = 0.867, sensitivity 76%/specificity 82%) [38] and kininogen-1 (AUC = 0.635, sensitivity 70%/specificity 66%) [39].

We performed ectopic expression of *SPON2* in colon cancer cell lines and found that the upregulated SPON2 induced an accelerated proliferation of the cells. This observation indicated that SPON2 might involve in the malignancy process of colon cancer.

In summary, our current analysis suggested that SPON2 might involve in the tumorigenesis and malignancy of CRC and SPON2 could be an independent diagnostic or prognostic biomarker of CRC.

## MATERIALS AND METHODS

### Cell lines and cell culture

Cell lines LoVo, Caco-2, Colo-320DM, HCT-116 and SW-620 were brought from the Cell Bank of Shanghai Institutes for Biological Sciences, China. LoVo and Colo-320DM cells were cultured in RPMI 1640 supplied with 10% FBS. Caco-2 cells were maintained in DMEM supplemented with 20% FBS. SW-620 cells were cultured in DMEM supplemented with 10% FBS, whereas HCT-116 and HT-29 cells were cultivated with McCoy's 5A medium (Gibco Life Technologies, Shanghai, China)/10% FBS. All culture media were supplemented with 1% penicillin/streptomycin (Hyclone Laboratories, China) and cells were all cultured in a humidified incubator at 37°C and 5% CO_2_.

### CRC tissue samples used for qRT-PCR analysis

Colon adenocarcinoma tissues and the matched normal mucosa counterparts were collected during surgery from 10 patients at the Shanghai Public Health Clinical Center, Fudan University, Shanghai, China. The adjacent normal colonic tissues were collected at the same time at least 5-cm away from the loci of cancerous tissues. The clinic specimens were histologically checked under microscopy by pathologic experts and information was recorded. The study was approved by the Clinical Research Ethics Committee of Fudan University.

### Antibodies

SPON2 antibody was purchased from Sigma-Aldrich (Shanghai, China). Antibody of GAPDH was purchased from CWBIOTECH, Beijing, China.

### Cellular protein extraction and western blot

Cultured cells were washed twice with cold PBS and lysed using SDS lysis buffer containing 50 mM Tris-HCl (pH 8.1), 1% SDS and 1 × proteinase inhibitor (Roche, Shanghai, China). The lysates were treated with 50 mM DTT and heated in boiling water for 15 min. After clearance with centrifugation, protein concentration of the supernatant was quantified using a bicinchoninic acid assay (BCA) protein assay kit (Beyotime, Haimen, China). Proteins were separated by 10% SDS-PAGE and transferred onto Immobilon-P Transfer membrane (Merck Millipore, Shanghai, China). Immunoblots were performed using standard protocols. Thermo Scientific SuperSignal West Femto Maximum Sensitivity Substrate (Thermo Scientific Pierce, Shanghai, China) was used to detect the signal of Western blot with a LAS-3000 imager (Fujifilm, China).

### Data mining using oncomine gene expression microarray datasets

*SPON2* gene expression was analyzed using microarray gene expression datasets deposited in Oncomine database (https://www.oncomine.org/resource/login.html). First, to address the differential expression of *SPON2* between colon cancer and normal tissues, a combined filters were applied to display the corresponding datasets. The Cancer Type was defined as Colorectal Cancer and Data Type was mRNA, whereas Analysis Type was Cancer vs Normal Analysis. To display and analyze datasets with survival-associated expression, an additional filter of Survival Status was applied, while the Analysis Type filter was removed. To analyze the relationship of *SPON2* expression with different clinicopathological parameters, only “mRNA” and “Colorectal Cancer” filters were applied to display as many as datasets for further analysis. The datasets passed the filters were then displayed and each dataset could be analyzed individually. The visualization of each dataset could be varied by changing the “group by” drop-down menu to shown different clinicopathological parameters. In case where there was more than one reporter (probe) available, the first reporter was selected by default. The expression values of *SPON2* gene were read from the displayed bar chart. These values were parsed into Excel and analyzed. Student's *t* test was used to calculate the significance. To performed χ^2^ test and Kaplan-Meier survival analysis, *SPON2* expression was classified as higher-than-median or lower-than-median groups.

### RNA extraction and qRT-PCR

Total cellular RNA was extracted using TRIzol reagent (Life Technologies-Invitrogen, Shanghai, China) according to the manufacturer's instructions. First strand cDNA was produced using the PrimeScript 1st Strand cDNA Synthesis Kit (Takara Bio Inc., Shanghai, China). qRT-PCR was performed using the IQ5 Real-Time PCR detection system (Bio-Rad, Shanghai, China) with SYBR green (Takara Bio Inc., Shanghai, China). The qRT-PCR primers of *SPON2* were spon2-F (AAGAACCAGTACGTCAGTAACGG) and spon2-R (CACAAACGAGACCAGCGAGT).

### IHC and scoring

IHC was performed with SPON2 antibody at a 1:50 dilution using two commercial TMAs (Catalog no. HCol-Ade180Sur-04 and HCol-Ade180CS-01, Shanghai Outdo Biotech, China). The first TMA consists of 90 pairs of CRC tissues and the normal mucosa counterparts. The surgical time was from July 2006 to May. 2007 and the follow-up information was available from November 2006 to Aug. 2013. The survival time was 3 ~85 months with a median survival time of 56 months. Follow-up records were unavailable for 7 cases. The second TMA contains 90 pairs of CRC tissues and the matched normal counterparts without follow-up information. The 90 CRC cases included 2 cases of grade I, 1 case of grade I-III, five cases of grade I-II, 59 cases of grade II, 17 cases of grade II-III and 6 cases of grade III, which were histologically checked and diagnosed by clinician experts. The clinicopathological information of all cases were publically available at the company website (http://www.superchip.com.cn/).

IHC analysis was performed according to an established protocol of Outdo Biotech. The immunostaining was developed using the substrate 3,3′-diaminobenzidine (DAB) and the nuclei were counterstained with hematoxylin. After staining, the chip was scanned using Scanscope XT (Aperio, Shanghai, China). To calculate the staining intensity, sample images of each spots was exported using Aperio ImageScope v. 12. The cancer stroma or useless regions in the images were blocked using the Image-Pro Plus before downstream analyses. Next, the images were subjected to measurement of IOD of the epithelial regions. The parameters for image segmentation and intensity calibration were adjusted with one images and were used for all images. Mean IOD of each image was calculated by dividing the IOD with the corresponding area. The mean IODs from different images of the same sample were averaged. The averaged IOD was used to represent the expression level of SPON2 in each sample. In addition to the automatic scanning method, a manual scoring was performed independently to classify the expressions into negative or positive group. Both scoring results were combined and compared and a final cutoff was determined for the negative or positive expression. Samples without follow-up information or with no visible epithelial cells were excluded from analysis.

### Statistics

Survival curves were calculated using the Kaplan-Meier algorism and Log-rank (Mantel-Cox) test with GraphPad Prism 6.01. The correlation between SPON2 (mRNA or protein) expression and the clinicopathological parameters of CRC patients were evaluated by χ^2^ test using PASW Statistics 18. The significance between the normal and cancerous samples was calculated using two-tailed Student's *t* test. A *p* value < 0.05 was considered as statistically significant. Univariate and multivariate statistics was performed with PASW Statistics 18 using enter and forward likelihood ratio method, respectively. A *p* value < 0.05 was accepted as significant with 95% confidence intervals.

### ELISA

Clinic plasma sample collection and ELISA experiments were performed as previously described [40, 41]. The plasma samples from 28 healthy individuals, 13 CRC patients, 26 GC patients, 7 prostate cancer patients and 6 benign prostatic hyperplasia were collected at the First Affiliated Hospital of Soochow University, Jiangsu, China. There were 30 CRC plasma samples collected at the Beijing Cancer Hospital, Beijing, China and 50 CRC plasma (post-surgery) were collected from the First Affiliated Hospital of Zhengzhou University, Henan, China. The CRC samples selected for analysis were adenocarcinomas restricted to the large intestine. The prostate cancer and GC samples were also adenocarcinomas. The healthy donors providing the plasma samples were all confirmed to be free from any disease by routine body examination. Informed consents were signed by patients. The analysis was permitted by the Clinical Research Ethics Committee of Fudan University. The plasma samples were kept at −80°C before use. The ELISA protocol was optimized and the plasma was diluted 10 fold with PBS (pH 7.2) before reaction. The ELISA kit (Cloud-Clone Corp) for SPON2 was purchased from the Wuhao, Inc., Shanghai, China. The concentration of SPON2 in the plasma was determined using the standard protein sample provided by the kit. The ROC curve analysis was performed using GraphPad Prism 6.0.1 and the optimal concentration of SPON2 was determined at the maximal sensitivity + specificity.

### Gene cloning and cell transfection

The full-length *SPON2* gene was amplified from cDNA previously prepared from human normal colonic fibroblast cell line CCD-18Co [40] and cloned into pcDNA 3.1/*myc*-His(−) A vector (Invitrogen-Life Technologies, Shanghai, China). The cloning primers used were spon2-F-NheI: CTAGCTAGCGCCTGCCGGGTGATGGAAAAC and spon2-R-HindIII: CCCAAGCTTGACGCAGTTATCAGGGACGC. DNA transfections were performed using Lipofectamine 2000 (Life Technologies - Invitrogen) with the aid of OPTI-MEM reduced serum media (Life Technologies - Invitrogen) according to the manufacturer's instructions. The cell culture medium was refreshed 6 hours after transfection.

### Cell migration assay

Cell migration was assessed using Transwell plates (Corning Inc., Shanghai, China) according to the manufacturer's instructions. Briefly, colon cancer cell lines were transfected with plasmids and were incubated in serum-free medium for 24 h. The transfected cells were transferred to the upper chamber of a Transwell plate. Next, 0.5 mL medium containing 10% FBS was added to the lower chamber as a chemoattractant. The cells were further cultured for 16-24 h at 37°C. The cells remained on the upper membrane surface were scraped off with cotton swabs. The cells migrated to the reverse surface of the membrane were stained with 0.1% crystal violet for 10 min. Residual dye was removed by washing with water for 30 seconds. The dye was dissolved with 33% acetic acid and the absorbance at 570 nm was detected using a spectrophotometer. Each experiment was performed in triplicate.

### Proliferation assay

After transfection, colon cancer cells were seeded in 96-well plates, where each time point had at least five replicates. The proliferation was measured using MTT (Sigma-Aldrich, Shanghai, China) method. The MTT transformed crystals were dissolved in DMSO. The absorbance of the dye at 490 nm was detected using an Epoch microplate spectrophotometer (BioTek).

## SUPPLEMENTARY TABLES





## Abbreviations

AUC: area under the curve
BCA: bicinchoninic acid assay
CI: confidence interval
CRC: colorectal cancer
DAB: 3,3′-diaminobenzidine
ECM: extracellular matrix
ELISA: Enzyme-linked immunosorbent assay
GAPDH: glyceraldehyde-3-phosphate dehydrogenase
HR: hazard ratio
IHC: immunohistochemistry
IOD: integrated optical density
ROC: receiver operating characteristic
SPON2: spondin-2
qRT-PCR: quantitative reverse-transcriptional PCR
RNAi: RNA interference
TMA: tissue microarray
